# Generative Adversarial Networks and Data Clustering for Likable Drone Design

**DOI:** 10.3390/s22176433

**Published:** 2022-08-26

**Authors:** Lee J. Yamin, Jessica R. Cauchard

**Affiliations:** Magic Lab, Department of Industrial Engineering and Management, Ben Gurion University of the Negev, P.O. Box 653, Beer-Sheva 8410501, Israel

**Keywords:** deep learning, data clustering, generative adversarial networks, human-drone interaction, drone design

## Abstract

Novel applications for human-drone interaction demand new design approaches, such as social drones that need to be perceived as likable by users. However, given the complexity of the likability perception process, gathering such design information from the interaction context is intricate. This work leverages deep learning-based techniques to generate novel likable drone images. We collected a drone image database (N=360) applicable for design research and assessed the drone’s likability ratings in a user study (N=379). We employed two clustering methodologies: 1. likability-based, which resulted in non-likable, neutral, and likable drone clusters; and 2. feature-based (VGG, PCA), which resulted in drone clusters characterized by visual similarity; both clustered using the K-means algorithm. A characterization process identified three drone features: colorfulness, animal-like representation, and emotional expressions through facial features, which affect drone likability, going beyond prior research. We used the likable drone cluster (N=122) for generating new images using StyleGAN2-ADA and addressed the dataset size limitation using specific configurations and transfer learning. Our results were mitigated due to the dataset size; thus, we illustrate the feasibility of our approach by generating new images using the original database. Our findings demonstrate the effectiveness of Generative Adversarial Networks (GANs) exploitation for drone design, and to the best of our knowledge, this work is the first to suggest GANs for such application.

## 1. Introduction

The last decade has seen a revolution in the field of robotics, with drones becoming ubiquitous in human environments [1,2]. Their usage is rapidly expanding, thus enabling novel applications. Specifically, in the field of Human-Drone Interaction (HDI) [3], we find novel applications for social and domestic drones, which have the potential to help people in their daily lives [4,5]. However, for this vision to become a reality, prior research has shown that the design of the drone itself needs to be adapted to its application to support users’ expectations [6]. In particular, for technology to be accepted in a social context, prior work has described the need for it to be perceived as likable [7]. However, given the complexity of the likability perception process, gathering such design information from the interaction context is intricate.

We here propose to leverage Deep Learning (DL)-based techniques, including Generative Adversarial Networks (GANs), to generate novel likable drone images in support of future researchers and manufacturers. While such methods are commonly used for generating synthetic data, recent work proposed applying them to the design of novel technologies [8]. In their work, Gan et al. [8] used aesthetic and emotional evaluations of social robots to generate novel effective designs of such robots. Their results led to a set of computer-generated images given to designers to finalize selected prototypes that human participants then validated. Motivated by their promising results, we explore a fully computational approach using StyleGAN2-ADA with the aim to generate new likable drone designs without human intervention.

In this work, we first collected a drone images database (N=360) tailored to consider drone design research needs. Each drone image was then rated for likability in an online user study (N=379), resulting in 13,965 individual drone image ratings, with at least 35 ratings per drone image. We opted to cluster the data in two ways, first using the likability ratings (likability-based clustering) and second using a fully computational approach (feature-based clustering) using a pre-trained VGG network for high dimensional feature extraction from the images and PCA for dimensionality reduction. Both clustering processes employ the K-means algorithm. We further describe a clustering characterization method for determining drone features that affect likability using visual design elements on the drones, as in [6]. This dual approach aimed to identify whether the current visual design elements identified in the literature appear exhaustive or if additional elements could be uncovered. A correlation between likability-based and feature-based clusters did not reveal additional visual features that may explain similarities or differences between drone clusters. Our next step was to use the cluster of likable drones using StyleGAN2-ADA to explore whether new likable drone designs could be generated. This exploration included experimenting with several parameters, specific settings, and five pre-trained networks for transfer learning. While this approach did not yield the expected results due to overfitting, we further illustrated the feasibility of our approach by generating new valid drone designs based on the original drone database.

The main contributions of the proposed work can be summarized as follows:

- Computational methods are effective for categorizing drone images based on human perception.
- Specific visual features on drones (colorfulness, animal-like representation, facial features with emotional expressions) are identified as markers of likability.
- New drone designs can be automatically generated using GANs.

The rest of the paper is organized as follows: Section 2 presents related work starting with the impact of drone design on HDI and following with background literature on the use of GANs. Section 3 describes the materials and methods used to explore likable drones and generate novel designs. It details how the drone image database was built, the data collection of likability ratings through a user study (N=379), the dual approach of likability-based and feature-based data clustering, and finally describes our selection and configurations of StyleGAN2-ADA for generating likable drone designs. Section 4 describes the results of our work, and Section 5 discusses them. Finally, Section 6 presents future work directions, and Section 7 the conclusions and lessons learned from this work.

## 2. Related Work

This section presents the state-of-the-art research in HDI and the impact of design on interaction with such technology, as well as background literature on GANs.

### 2.1. Impact of Drone Design on Human-Drone Interaction

Personal drones were initially used for applications such as taking photos and videos, yet their high availability and affordability helped increase the number and type of scenarios of use [9]. Recently, Herdel et al. [3] presented a holistic view of domains and applications of use that are described, studied, and envisioned in the HDI body of work, where 16 domains and over 100 applications were identified where drones and people interact together. For this rich range of domains (e.g., entertainment, companionship, communication, assistance), beyond delivery services [10], drones may play a role as sports coaches [11], tour guides [12,13], and in playing games with people [6,14,15]. They can also support people by helping them navigate [16,17,18], walking them home safely at night [19] or assisting elderly populations [5,20]. In this wide range of use cases, drones interact with users [21] or bystanders [22] alike. The use of drones with many applications in diverse domains, in private or public environments, with various end-users outlines many forms of interaction. Baytaş et al. [23] defined autonomous drones operating in public spaces as social drones as some form of social interaction is unavoidable. As such, it is critical to elevate the human–drone interaction. Prior work on interaction with drones explored several feedback mechanisms such as using LEDs to convey intent [24], a screen [25], a projector to display a map [12], or using the flight path to convey intent and emotions [26,27,28,29]. The drone’s emotional state was shown to be effective as a form of feedback to the user. Herdel et al. [29] explored the use of facial features to represent emotions on social drones and demonstrated that people could recognize different emotional states (i.e., joy, sadness, fear, anger, and surprise) and be emotionally affected by the drone.

Recent work has emphasized aesthetics and user preference-based design to enhance communication and social acceptability. The drone in its design may convey some information regarding its intent, functions, and capabilities. We find that for ground robotics, the design of the robot itself, its shape, size, color, and facial attributes influence how people perceive them [30,31]. Prior research has been working toward design guidelines for social drones suitable for interaction and companionship. Kim et al. [32] proposed that an ideal companion drone should present “adorability” features. Chang et al. [33] found that the drone’s color, size, and shape seem to influence how it is perceived. Yeh et al. [34] proposed a blue oval-shaped drone and discussed how a tablet could be used to display a “friendly face”. Karjalainen et al. [35] investigated several features and found that emotional characteristics were desirable, and they also suggested that the drone’s appearance should be a round shape with a face. It was shown that robots without a face are perceived as less sociable and amiable compared to robots with a face [36]. Herdel et al. [29] proposed to add facial expressions onto a drone’s body to enable its social perception by humans. The above literature highlights the growing interest in drone design for interaction.

**Figure 1:**
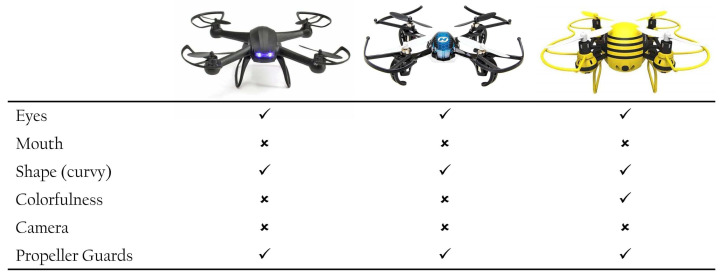
An example of drones labeled following Wojciechowska et al. [6] method of encoding. Despite their different visual appearances, the drones have in common: eyes, propeller guards, and curvy lines, and none feature a mouth or visible camera. The encoding only differs in colorfulness; yet, it does not express core differences in the drones as perceived by the human visual system.

Wojciechowska et al. [6] explored how the drone’s physical features affect how it is perceived across eight dimensions of perception, i.e., animal-likeness, friendliness, intelligence, trustworthiness, age, gender, interaction, and likability. They defined seven physical features in their encoding, i.e., colorfulness, shape, facial features as in mouth, eyes and their colorfulness, visible camera, and propeller guards. They quantified this effect with a regression model and presented a set of design guidelines for future drones. For example, likability in drones is associated with eyes, a visible camera, curvy lines, no propeller guards, and a colorful body. However, encoding such design information in a few variables is questionable due to the complexity of the human perception process. Drones could be encoded the same or similar while presenting highly different designs in our visual system. An example of this concern is presented in Figure 1. For the purpose of designing new drones, encoding such visual information is limited compared to the human visual system.

As the interest in drones in social contexts is increasing, in this work, we focus on the perceived likability of drones. The likability of drones has shown prior interest [6] and holds a common measurement tool [37]. Moreover, as drones evolve as technology and more applications are expected, it is essential to define design guidelines for future likable drones continuously. We explore whether new likable drone designs can be automatically generated for drone designers and manufacturers means. Recent advances in DL, specifically in synthetic data generation using GANs, can be valuable for HDI research. Such a method does not require feature encoding and may perceive visual stimuli meaningfully. In the next section, we will present the literature background of GANs and state-of-the-art networks for data generation.

### 2.2. Generative Adversarial Networks

Computer Vision (CV) has evolved over the years to improve computer systems’ capability to perceive visual stimuli meaningfully [38]. DL was developed to improve CV techniques and manifested outstanding performance for many applications, including image generation. Specifically, the realization of GANs has led to state-of-the-art results [39].

GANs were first introduced in 2014 by Goodfellow et al. [40] to generate better synthetic images than previous generative models, e.g., variational autoencoders [41]. The generative framework comprises two antagonistic models; the generator, trained to generate new data samples, and the discriminator, which tries to classify samples as either real or synthetic. The two models improve each other adversarially until the discriminator can no longer distinguish between real and synthetic data. After the training process, GANs are capable of generating realistic images from randomly sampled latent spaces.

In recent years, extensive research efforts have contributed to improving the performance of GANs. The Deep Convolutional GAN (DCGAN) improved the resolution and image quality results obtained with original GANs [42]. The WGAN proposed to use the Wasserstein distance to replace the Jensen–Shannon distance, which can better measure the difference between the two data distributions [43]. ProGAN introduced an innovative approach to progressively increase the generator and discriminator for improved quality, stability, and variation [44]. The training process starts with low-resolution images. Progressively, the resolution of the images is doubled, and new layers are added simultaneously, increasing the spatial resolution. One of the advantages of this type of architecture is that the neural network initially uncovers the basic structure of the images and progressively moves its attention to the finer details.

The realization of ProGAN has given life to StyleGAN [45]. The main feature of StyleGAN, which distinguishes this architecture from previous work, is its style-based generator which allows automatic separation of stochastic variations (e.g., position) from high-level features (e.g., pose). StyleGAN2 [46] is StyleGAN’s subsequent improved implementation where its network modifies the generator normalization to eliminate artifacts from the generated images. StyleGAN2 allows the generation of high-quality and high-resolution images; however, one challenge is to train this type of network with limited data. Training using a small dataset typically leads to discriminator overfitting, causing training to diverge. To address this problem, StyleGAN2 was enhanced using Adaptive Discriminator Augmentation (ADA), namely StyleGAN2-ADA [47]. This technique corresponds to an analogy of putting distorting goggles on the discriminator and asking the generator to produce samples that cannot be distinguished from the training set when viewed through the goggles, such that the generator does not learn the augmented distribution (non-leaking). This way, StyleGAN2-ADA allows converting noninvertible data augmentation to invertible transformations using an adaptive augmentation probability. The authors demonstrate the feasibility of using limited datasets to obtain reliable stabilized training, vastly improving the resulting quality of StyleGAN2, and establishing the significance of transfer learning in such conditions. Transfer learning reduces training data requirements by starting from a model trained using another dataset instead of random initialization. Transfer learning was shown to give significantly better results than from scratch training, and its success depends primarily on the diversity of the source dataset instead of the similarity between subjects [47]. More recently, StyleGAN3 was introduced, addressing the synthesis process of typical GANs, which depend on absolute pixel coordinates causing detail to appear to be glued to image coordinates instead of the surfaces of depicted objects [48]. The core innovation lies in aliasing the generator network with small architectural changes that guarantee that unwanted information cannot leak into the hierarchical synthesis process. StyleGAN3 establishes generative models better suited for video and animation.

As described above, this research explores the use of GANs to generate likable drone designs. To the best of our knowledge, this work is the first to suggest such an approach for the generation of aerial robotic technologies. The following section describes our drone images database as its design will impact the selection of GAN. We further detail in Section 3.4.1 which type of GAN is best suited for this research and why.

## 3. Materials and Methods

**Figure 2:**
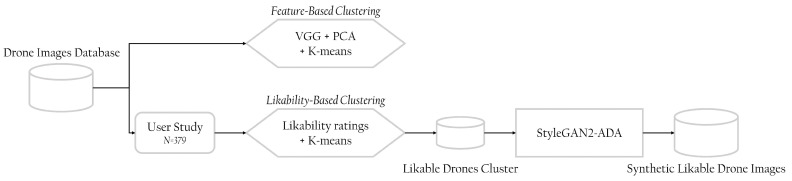
Workflow of the materials and methods proposed in this work; starting with a drone images database, we run a user study (N=379) to collect likability ratings of drones. We cluster the data using two methodologies: likability-based and feature-based. We then focus on the likable drones cluster to generate synthetic ones using StyleGAN2-ADA.

This section presents the methodology for exploring likable drone design generation. It first introduces our collected drone images database, followed by the questionnaire design and user study for collecting likability ratings for the drone database. Then, it describes our techniques for likability-based and features-based clustering. Finally, it presents our method for generating likable drone images. Figure 2 summarizes the suggested methodology in a workflow.

### 3.1. Drone Images Database

The first step was to identify a drone database suitable for DL-based methods that can be validated for likability by human participants. Recent work has produced a dataset of 63 drone images [6] with specific characteristics for design research, such as having all drone images at scale, not including other objects or text in the images, having the drones on a light unicolor background, and propellers at rest. To the best of our knowledge, there is no other dataset of drone images available for design research and perception studies. While this dataset is well suited for this research, it is limited for DL applications and requires further data collection. As a starting point, we used 58 images from this dataset [6] as a preliminary database (*Note: 5 drone images were not suitable, such as a white drone on a colorful background, and as such, were not included in our database, resulting in 58 out of 63 drones*). Our work also focuses exclusively on commercial quadcopters.

**Figure 3:**
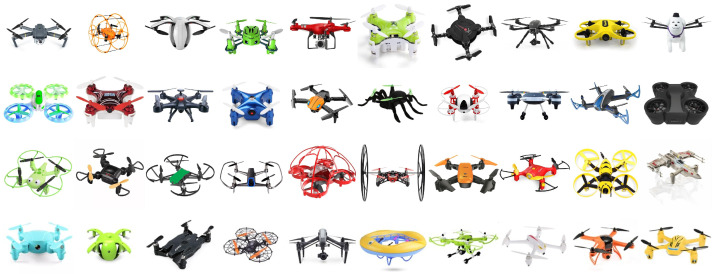
A randomly selected sample of the drone images database to illustrate the diversity in drone design in shape, color, guards, guards shape, guards color, camera, facial features (i.e., eyes, mouth) and expressions, resemblance of objects (e.g., apple, aircraft), or animals (e.g., bee, dog, spider).

We collected drone images in July and August 2021 using a web-scraping algorithm for predefined keywords (e.g., “drone”, “drone model”, and “quadcopter”) in multiple languages including English, Hebrew, and Chinese. The algorithm automatically excluded low-resolution images and images with a colorful background. This data collection resulted in a large number of drone images, for which we then conducted the procedure described when generating the first dataset [6] to narrow down the database to suitable images only. This process led to a collection of 302 drone images, resulting in a comprehensive database of 360 drone images. Out of these, 24 drone images (12 pairs) presented the same drone shown at a different angle. We kept these images for the training of our GAN. For the user study, we randomly selected one of each pair as both would be perceived equivalently for likability. Images were cropped, reduced to 256×256 in size, and padding was added when needed. Figure 3 shows a selected sample of the database to emphasize the diversity of drone designs in color, shape, physical features such as guards and camera, facial expressions, and resemblance to different objects or animals. The approach of using a static image database is based on prior research [6,49,50].

### 3.2. Data Collection

This section describes the questionnaire design and the user study to measure the likability of drones in the database.

#### 3.2.1. Questionnaire Design

We aimed to measure the likability of each drone image. Several questionnaires exist in the literature. While Wojciechowska et al. [6] used a continuous rating scale of 0 to 100 [6], we opted for a more common measurement tool of likability in human-robot interaction, namely the God-speed III questionnaire, which has shown sufficient internal consistency reliability [37]. It measures the likability of robots via five questions using a 5-point semantic differential scale for each question: “Dislike/Like”; “Unfriendly/Friendly”; “Unkind/Kind”; “Unpleasant/Pleasent”; and “Awful/Nice”.

The questionnaire also included demographic questions related to participants’ age, gender, and education level, as well as questions about previous experience with drones.

#### 3.2.2. User Study

The questionnaire was distributed via Amazon Mechanical Turk (mTurk), a crowd-sourcing platform allowing workers over 18 years old to complete online tasks for pay. The mTurk workers were sampled from the United States, with an excellent performance history, HIT approval rate ≥ 97, and an approved number of HITs ≥ 50. Participants were asked to read and sign an anonymized electronic consent form. They were then presented with 30 randomized drone images and the five likability questions described above. Halfway through the study, participants were asked to answer a simple control question to check their attention span. We disqualified 17 questionnaires for which the control question was incorrect or for which the same answer was given to all questions. A total of 379 approved volunteers were sampled for this study. The participant pool comprised 156 females, 221 males, 1 non-binary, and 1 unknown gender, from 18 to 65+ years old, with most participants in the age ranges of 25–34 (35%) and 35–44 (37%) years old. 95% of participants had a college degree or higher, and the majority (94%) reported having seen a drone before participating in the study. On average, questionnaires were completed in 15 min 20 s.

The data collection resulted in 13,965 drone likability ratings with a minimum of 35 individual ratings for each drone. Drone likability values were calculated as medians of individual answers to the average value of the five scales.

### 3.3. Data Clustering

We opted to cluster the data from two different perspectives. The first consisted of a likability-based clustering method, which consisted of clustering the data based on likability ratings and characterizing the clusters using visual design elements of the drones, based on prior research. The second took a computational approach and used a fully automated feature-based clustering method. The goal of this dual approach was to identify whether the current visual design elements identified in the literature appear exhaustive or if additional elements could be uncovered. A correlation between likability-based and feature-based clusters may shed light on more design features that may affect the human likability perception of drones.

#### 3.3.1. Likability-Based Clustering Methodology

Our goal was to identify drone clusters using the likability ratings. We employed a two-step characterization process, where we first exploited the drone likability ratings into clusters, then compared the clusters based on the design characteristics of the drones. This section first describes the likability-based drone clusters that were formed using K-means. It then describes the cluster’s characterization process.

In the work of Wojciechowska et al. [6], two thresholds were presented for unlikable vs. likable at respective values of 30 and 70 on a scale of 0–100. However, it was not specified how this threshold was established, and as such, we sought a more validated method of clustering. We employed K-means [51], a popular algorithm for its simplicity and efficiency [52]. The main element of the algorithm is based on expectation-maximization, and the quality of the cluster assignments is determined by computing the Sum of the Squared Error (SSE). The objective of the K-means algorithm is to minimize this value. A primary step for K-means is to determine *K*, the number of clusters. A common technique for setting *K* is using the elbow method [53], which includes running the K-means algorithm for several iterations where, in each iteration, *K* is incremented, and the SSE is recorded. The elbow point represents the SSE convergence and a reasonable trade-off between error and the number of clusters. We clustered the likability ratings using the K-means algorithm using the median likability values of each drone. Based on our domain knowledge, we expected three clusters (K=3) representing non-likable, neutral, and likable drones. We validated this optimal number of clusters using the elbow method [53].

Following this, our next step was to determine the underlying features that characterize the drone clusters. With this data, one of the challenges we faced was to identify specific features in the images that may affect the likability of the drone. Since prior research demonstrated a set of design elements on drones that affect drone perception (including likability) [6] we ran the analysis of cluster characteristics based on their findings. We then determined the features of clusters based upon the following characteristics: colorfulness, shape, propeller guards, camera, facial features, and animal-like representation. We also chose to explore drone facial expressions that convey emotions, following recent findings by Herdel et al. [29], identified as Joy, Sadness, Fear, Anger, and Surprise. This labeling procedure was conducted manually. The resulting histograms or numbers of drones with specific characteristics were then processed for characterization by the research team.

#### 3.3.2. Feature-Based Clustering Methodology

Our goal here was to identify drone clusters using raw image features. Numerous CV-based techniques emulate the human visual system, including DL-based approaches. We chose the Visual Geometry Group (VGG), a classical deep convolutional neural network architecture [54]. The VGG architecture is the basis of ground-breaking object recognition models [55], and different VGG networks are referenced with their number of layers, i.e., VGG16 and VGG19. Following their outstanding performance, VGG networks are used for image feature extraction. We used a pre-trained VGG16 network [55]. The features extracted are of high dimensionality, which is computationally expensive for further operations. We utilized the commonly used technique for dimensionality reduction, namely Principal Component Analysis (PCA) [56]. Once the features were identified, we again used the K-means algorithm to cluster the drones based on visual similarity in images. These clusters will characterize drones with visual similarities and differences, and correlation with the likability-based clusters may help determine new relevant design features. The following section presents how the resulting clusters were used to generate new likable drones.

### 3.4. Data Generation

Given the increasing number of applications in the space of social robotics with drones [3], we explore if new likable drone images can be generated automatically. Since recent progress in computational methods enables the generation of new images (also known as synthetic data), we propose to apply such a method to our data. This section first describes our choice to use GANs, specifically StyleGAN2-ADA, to generate new images of likable drones. It then describes the configurations we experimented with, considering the size limitations of our data. Further, it explains the evaluation metric used to evaluate the best configuration. Finally, it presents our training parameter settings.

#### 3.4.1. StyleGAN2-ADA for Generating Likable Drones

The StyleGAN family is the current state-of-the-art GAN in generating high-definition synthetic images. In this work, we used the StyleGAN2 network [46] and trained it using the generated cluster of likable drones (see Section 3.3.1), comprised of 122 drone images. This size dataset is considered limited for GANs, and as such, there is a need to explore specific configurations for training control. We employed the ADA operation that improves the StyleGAN2 performance for limited datasets, namely StyleGAN2-ADA [47]. Although StyleGAN3 [48] presents a more contemporary architecture, also consisting of the ADA enhancement, it was shown that its generator is comparable to StyleGAN2 for images while being computationally heavier. Therefore, we opted for StyleGAN2-ADA.

#### 3.4.2. StyleGAN2-ADA Configuration and Transfer Learning

Besides ADA, additional techniques are applicable to control training with limited datasets. For instance, a horizontal flip is a highly known augmentation for *x*-flips, referred to as mirror [47]. Given that the aspect position of the drone did not appear to affect its perception in terms of likability, we decided to use this operation, resulting in an enhanced training dataset.

While doubled, the size of our training dataset is yet limited, and we opted to apply transfer learning. Transfer learning, or knowledge transfer, is a Machine Learning (ML) method where knowledge gained during training in one setting is exploited to improve generalization in another [57]. Transfer learning reduces the training data requirements by starting from a model trained using a different dataset instead of random initialization. Prior work has explored this in the context of GANs [58,59,60], and Karras et al. [47] examined several transfer learning setups with ADA in limited data scenarios. Their results showed that transfer learning gives significantly better results than from-scratch training and that its success depends primarily on the diversity of the source dataset instead of the similarity between settings.

To find the best fit in terms of source network, we used multiple official pre-trained networks for images at 256×256 resolution, publicly available in the StyleGAN2-ADA PyTorch official implementation repository [47]. We selected networks that were trained on diverse datasets (i.e., human faces and cats) and introduce different dataset sizes and variances (e.g., CelebA-HQ-30k [61], LSUN CAT-1k [62], LSUN CAT-200k [63], FFHQ-10k [64], FFHQ-140k [65]). This investigation to determine the best training starting point is crucial as it may considerably affect the performance in generating drone images. As a baseline, we also trained from scratch (i.e., without transfer learning). The following subsection describes our chosen evaluation metric for GANs.

#### 3.4.3. GAN Evaluation Metrics

Multiple known metrics assess the quality of images generated by GANs. The current most standard metric is the Fréchet Inception Distance (FID) [66], which is calculated by computing the Fréchet Distance between two distributions of the real images and of the generated ones, fitted to feature representations of a pre-trained InceptionV3 network [67]. However, in small datasets, the FID is not an ideal metric because it becomes dominated by the inherent bias when the number of real images is insufficient [47]. The Kernel Inception Distance (KID) is a subsequent measure of GAN convergence [68], which is calculated using the squared maximum mean discrepancy between inception representations of the two distributions. The estimator has no bias or a small variance and is computationally faster than the FID [47]. A lower KID value shows a better generation of synthetic images. In the next section, we summarize the training parameters.

#### 3.4.4. Training Parameter Settings

The StyleGAN2 variants were trained using the original NVIDIA implementation on a computer with a Windows operating system with a single NVIDIA GeForce RTX 2080 Ti. We used the official StyleGAN2-ADA PyTorch implementation [47]. All images are with a resolution of 256×256. We enabled all available augmentations for ADA and set the mirror configuration to 1. The training of GANs includes the $R_{1}$ regularization, a regularization technique and gradient penalty [69]. Karras et al. [47] postulated that the best choice for the $R_{1}$ regularization weight γ is highly dependent on the dataset, suggesting experimenting with different values. Through trials and errors, we determined our data’s optimal $R_{1}$ weight to be γ=60 and set it accordingly. Other network parameters and loss function settings have not been modified and follow the original StyleGAN2-ADA implementation. We compared configurations between the five pre-trained networks used for transfer learning and between the baseline. Each run was set for 72 h. We evaluated the performance of each configuration using the KID metric. The next section presents the results of the data analysis and this data generation.

## 4. Results

This section describes the results of our work. We first present the user study results related to the drones’ likability ratings, followed by the likability-based and feature-based clusters analysis. Then, we report the results of StyleGAN2-ADA for generating likable drone designs.

### 4.1. Likability of Drones

**Figure 4:**
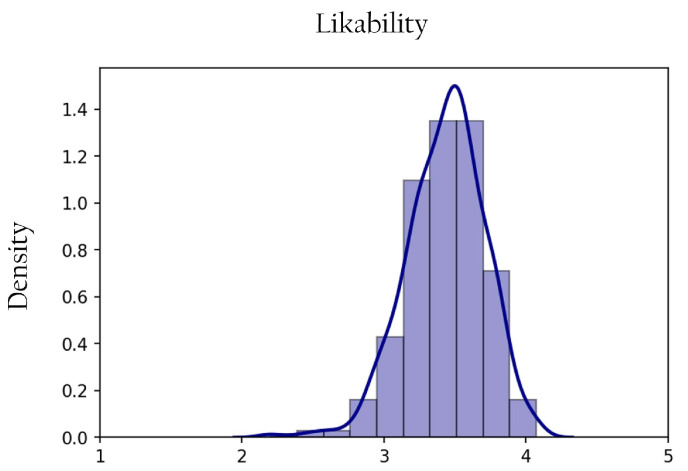
Histogram and corresponding probability density function of likability ratings. The likability scale is from 1 to 5 and the likability ratings range between 2.20–4.07 (μ=3.43,sd=0.05).

**Figure 5:**
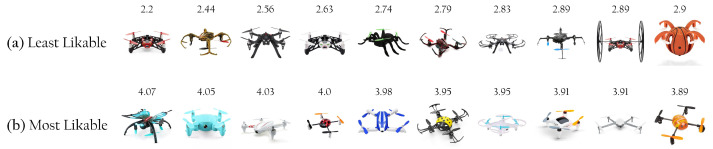
The ten lowest and ten highest ratings on the likability spectrum: (**a**) least likable and (**b**) most likable drones. The likability ratings are presented above each drone image.

The values of likability ranged between 2.20 and 4.07 out of 5 (μ=3.43,sd=0.05). Figure 4 presents the histogram of likability values and the corresponding probability density function. For reference, Figure 5 shows the ten least (a) and the ten most (b) likable drones, with the corresponding likability ratings.

### 4.2. Likability-Based Clustering

**Figure 6:**
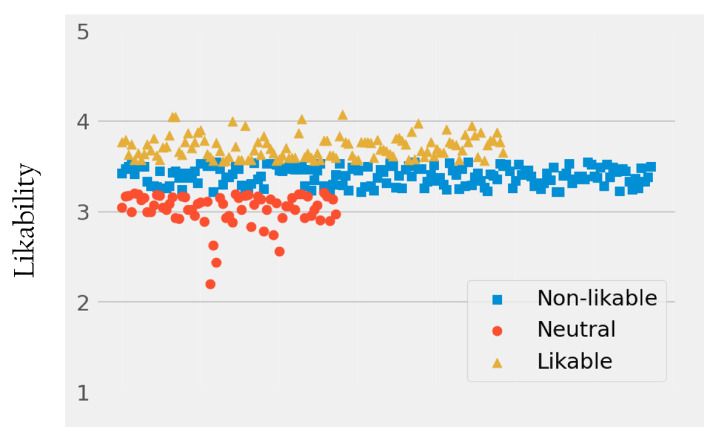
Visualization of the three likability-based clusters: non-likable (N=69), neutral (N=169), and likable (N=122) drone clusters.

The elbow method yielded the optimal number of clusters as three (K=3), which fits our expectation. Based on the ranging values of each cluster, we could refer to each with our domain knowledge, representing: non-likable (N=69), neutral (N=169), and likable drones (N=122). Accordingly, most drones are perceived as neutral in terms of likability, and there are more likable drones than non-likable drones (see Figure 6).

### 4.3. Likability-Based Cluster Characterization

This subsection reports on the results of the likability-based cluster characterization, which was used to determine the differentiating characteristics between clusters. We first present the three most relevant characteristics: colorfulness, animal-like representation, and emotional expressions of facial features. We end with a summary of the results yielded by the additional characteristics that were investigated.

#### 4.3.1. Drone Colorfulness

**Figure 7:**
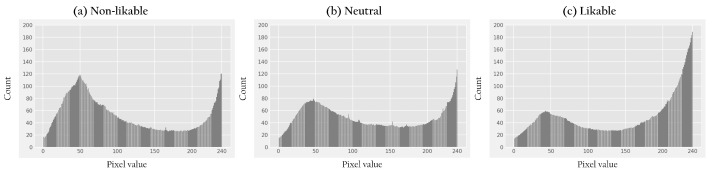
Histograms of pixel values of drone image clusters in (**a**) non-likable, (**b**) neutral, and (**c**) likable. The likable cluster (**c**) demonstrates brighter colors than the other clusters, and the darkest colors characterize the non-likable cluster (**a**). The pixel values presented are 0–240 to exclude the light background common for all clusters and comparison clarity. The values were weighted as 29.9%, 58.7%, and 11.4% for the red, green, and blue channels, respectively [70].

**Figure 8:**
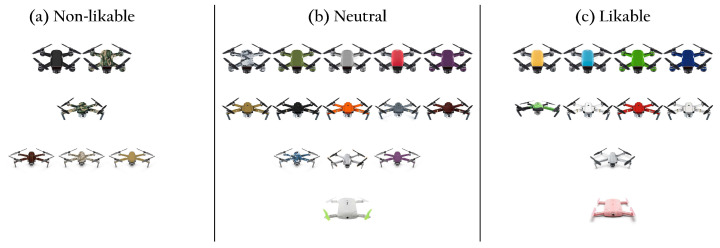
Drone colorfulness effect on perceived likability. Several instances of identical drone models with distinct colors were clustered differently. Non-likable drones (**a**) feature darker colors and military patterns. Drones with the same pattern in a different color are clustered as neutral (**b**), and likable drones (**c**) present brighter colors.

The likable drones cluster is characterized by brighter colors (higher pixel values) and the non-likable drones cluster by darker colors (lower pixel values), compared to the other two clusters, as can be seen in the pixel values histograms in Figure 7 (computed as the weight of 29.9%, 58.7%, and 11.4% for the R, G, B channels, respectively [70]). As such, we find that color plays a role in likability perception. These results further suggest that brighter and more intense colored drones are perceived as more likable and that darker drones are perceived as less likable. In addition, we find multiple instances of drones that differ only in their color that were rated and clustered differently (see Figure 8). We further find that drones with color patterns corresponding to armed-forces associations were perceived as non-likable. We found, for instance, two drones with such patterns but in blue and gray color (instead of green or brown), and these were clustered in the neutral-likability cluster. A specific color could not be identified to characterize a cluster.

#### 4.3.2. Animal-like Representation

**Figure 9:**
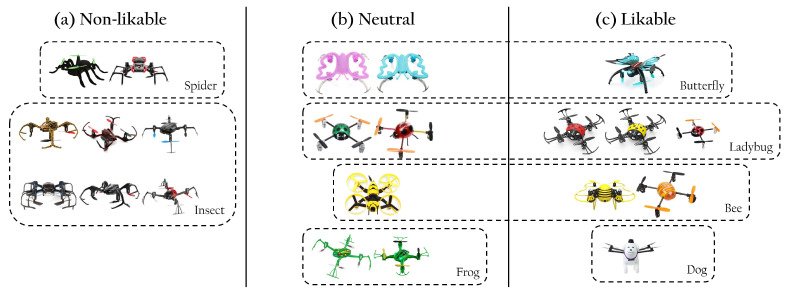
Example of drones designed with animal-like characteristics. Undesirable bugs and insects (e.g., spiders) are clustered in the non-likable cluster (**a**), while desirable ones (e.g., butterflies, ladybugs, bees) are clustered in the neutral (**b**) and likable (**c**) clusters. Frog designs appeared in the neutral cluster and the unique dog drone in the likable cluster.

The database comprises drones designed to resemble animals (e.g., bugs, insects, dogs). We found that different types of animals appeared in different clusters (see Figure 9). Indeed, drones designed as bugs with negative connotations (e.g., spiders, insects) are clustered in the non-likable cluster, while drones designed as insects with positive connotations (e.g., butterflies, ladybugs, or bees) are clustered in the neutral and likable clusters. Frog designs appeared in the neutral cluster and the unique dog drone in the likable cluster.

#### 4.3.3. Facial Features and Emotional Expressions

**Figure 10:**
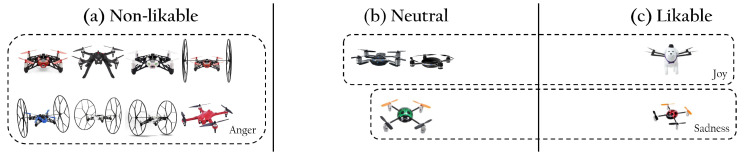
Drones designed with facial expressions of anger are clustered as non-likable (**a**); while expressions of joy and sadness are clustered in both the neutral (**b**) and likable (**c**) drone clusters.

The database comprises drones with characteristics that can be understood as facial features (e.g., eyes or mouth). We find drones with facial features in all clusters, and as such, this characteristic alone did not allow for characterizing differences between clusters. We then identified facial expressions of emotion. Three emotions were identified in our database: joy, sadness, and anger. We found that drones presenting emotional expressions of joy and sadness were clustered in the neutral and likable clusters, while drones with emotional expressions of anger were clustered as non-likable (see Figure 10).

#### 4.3.4. Additional Characteristics

Our analysis did not uncover a way to characterize the clusters based on the additional tested characteristics. The shape of the drone was labeled as either straight or curvy. We report the overall percentage of curvy drones with the database: 42%, and within clusters with 45% in the non-likable, 39% in the neutral, and 44% in the likable drone cluster. We labeled for the presence of a visible camera and report the overall percentage of drones with a camera: 50%, and within clusters: 33% in the non-likable, 52% in the neutral, and 55% in the likable drone cluster. We labeled for the presence of propeller guards and report the overall percentage of drones with propeller guards: 37%, and within clusters: 45% in the non-likable, 66% in the neutral, and 69% in the likable cluster.

### 4.4. Feature-Based Clustering

We present the clustering results based on visual similarities between drone images, using a pre-trained VGG16 network, PCA, and K-means to identify potential new design elements that may affect drone likability. The algorithm of the elbow method yielded the optimal number of clusters as four (K=4). The clusters, respectively, included: 49, 71, 99, and 141 drones. We then checked whether the feature-based clusters correlated with the likability-based clusters, and based on a Chi-Square test, we did not find a correlation ($\chi^{2}=49.34$, df=6, p<0.001). We ran the K-means algorithm a second time with K=3 and obtained three clusters, each comprising: 73, 102, and 185 drones. Again, these feature-based clusters did not show correlation with the likability-based clusters, Chi-Square test ($\chi^{2}=39.99$, df=4, p<0.001). Since no correlations were discovered, we could not identify additional design elements in drone design that may affect their likability.

### 4.5. Generating Likable Drones Using StyleGAN2-ADA

This subsection presents the results of generating likable drone images using StyleGAN2-ADA. First, we report on the performance of the five pre-trained network configurations for transfer learning and the baseline of training from scratch. We then present the selected performing configuration and report on the likable drone generation.

**Figure 11:**
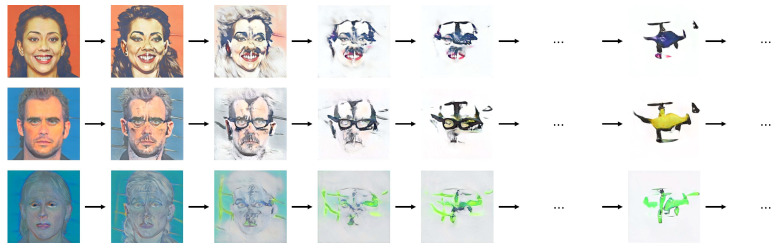
We illustrate the output of our transfer learning process using the CelebA-HQ-30k pre-trained network. As presented on the left, the network initially generated human faces and started generating likable drone images during training.

Table 1 presents the KID values for each configuration, and the time collapsed in hours until the optimal KID value was reached. A lower KID value shows a better generation of synthetic images; thus, starting training with the pre-trained CelebA-HQ-30k is best for our data. For reference, Figure 11 illustrates the process of transfer learning from the CelebA-HQ-30k pre-trained network to the generation of likable drone images.

**Figure 12:**
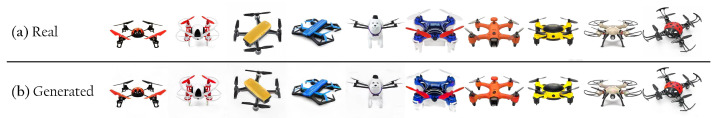
Real drone images (**a**) from the likable cluster above their corresponding drone generated images (**b**). The generated images overfit the drone images from the likable cluster. The overfitted generated images show high-level features that are unique to specific drone models. The differing orientation of drones is a result of the mirror operation.

**Figure 13:**
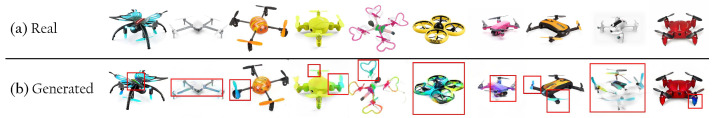
Real drone images from the likable cluster (**a**) above their corresponding drone generated images (**b**). The generated images present existing physical features colored in blue and blue color splutters (marked in red rectangles). We observed this phenomenon for the blue color exclusively.

We describe the results of the StyleGAN2-ADA network trained according to the previously detailed parameters with the configuration of the pre-trained CelebA-HQ-30k for transfer learning. Figure 12 presents generated likable drone images. However, the image illustrates the network’s overfitting where generated images fit the real images in the likable drone cluster. This phenomenon is referred to as mode collapse, where the generator fails to learn the distribution and is present for all drone models in the likable cluster. Interestingly, the generated images show specific high-level visual features, which are unique to the drone models generated. We further find multiple instances where generated images present artifacts or features in the color blue (see Figure 13). This phenomenon has been detected specifically for the color blue, while other colors were not witnessed.

While these results are promising, they are mitigated by the dataset size. As such, we further discuss these findings and demonstrate the feasibility of our approach by incorporating the entire drone images database to generate novel drone designs in Section 5.2.

## 5. Discussion

We discuss the use of computational methods for future designs, how drones can be generated using StyleGAN2-ADA, and present design guidelines for likable drones.

### 5.1. Using Computational Methods for Future Designs

In this work, we proposed an ML-based clustering method for drones using likability ratings gathered in a user study (N=379). Using the **K-means algorithm allowed clustering drones efficiently and systematically** without human intervention. This approach resulted in the drone images being clustered into: non-likable, neutral, and likable drone clusters. This result validated and extended prior findings in the human–drone interaction literature [6] that described that **drones can be classified in terms of likability** with lower and higher ends. We then characterized the clusters based on elements of drone designs established in prior work and found three characteristics: colorfulness, animal-like representation, and facial expressions of emotions, which could differentiate the clusters. These are further discussed in Section 5.3.

Next, we explored additional visual elements in drone designs that may not have yet been uncovered by prior work. To do so, we used a DL-based feature-based clustering method (VGG, PCA, K-means), which clusters based on visual similarity between images and does not rely on labeling, and yielded four clusters of drones. We then correlated the drone clusters between the likability-based and feature-based methods. These correlation results were inconclusive, and as such, we could not identify new drone features. Yet, this work shows that **drones can be visually differentiated using computational methods**.

While our computational methods are novel in this space, prior work by Gan et al. [8] proposed the use of DL-based methods (DCGAN) to generate novel design images of social robots. In their work, aesthetic and emotional evaluations were conducted by humans on existing images and were used as a base to generate novel effective designs that meet the aesthetic and emotional needs of customers. Their work showed a promising approach but required the intervention of designers, in addition to the computational methods, to generate viable designs. Moreover, their approach requires a larger dataset than currently available for drones, a more recent technology than ground robots. Instead, **our approach targets a smaller dataset size and enables a fully computational approach using StyleGAN2-ADA to generate new likable drone designs**.

### 5.2. Generating Drones Using StyleGAN2-ADA

To generate novel drone images, we used GANs, a fully automated method that does not require any prior feature-based extraction. The resulting generated images of likable drones were high-quality images with high-level features of each drone. Although, these generated images illustrated overfitting to the training data (see Figure 12). While our results are promising, they are limited by the dataset size consisting of 122 likable drones only and, accordingly, 244 training images after applying the mirror transformation. Given the novelty of this technology, especially for social applications, we expect that much time will be needed for this database to grow. As such, the field definitely needs support in generating new drone designs. Here, we demonstrate the **feasibility of our suggested approach by generating drone images based on the original drone images database**.

**Figure 14:**
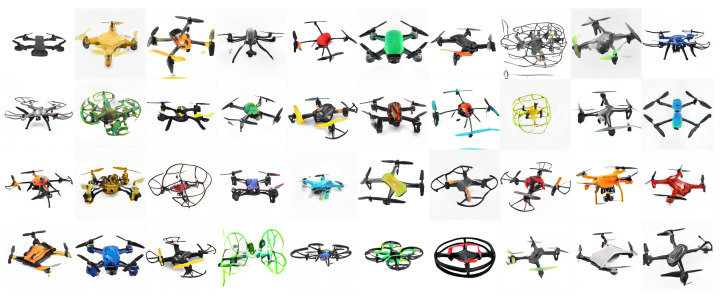
Drone images generated by the StyleGAN2-ADA network, trained on the full drone images database of 360 images. The results exhibit high-quality images with high-level features and high diversity. This highlight the feasibility of our suggested approach to future technology design.

We used the same training parameters settings with 360 drone images (i.e., 720 after the mirroring operation). This procedure resulted in generated drone images with once again, high-quality and high-level features for each drone. Moreover, **this yielded images with novel designs that do not exist in the database**. Figure 14 shows the diversity of the new drone models and colors generated by our approach using the entire database. This result highlights the **significance of the training dataset size and establishes the feasibility of the suggested methodology for future research** with more extensive databases. These automatically generated images of novel drone designs can support the work of drone designers and manufacturers, as well as the HDI research community. Based on our findings, we propose design guidelines for future likable drones.

### 5.3. Design Guidelines for Likable Drones

As the drone market expands, we envision drones will be built and designed for specific applications and usage scenarios. The results of our study follow and go beyond prior research in HDI to inform future drone designs to elicit likability in users.

Our results indicate that the **drone color plays a role in likability perception**. Brighter and more intense colored drones are perceived as more likable, while darker drones are perceived as less likable. This finding is on par with prior work that showed a positive trend between colorfulness and likability [6]. Moreover, drones with color patterns corresponding to armed-forces associations were clustered in the non-likable cluster. Furthermore, generated images by the StyleGAN2-ADA suggest that **the color blue characterizes the likable drones cluster**. This finding supports prior findings [34].

Prior work demonstrates a positive correlation between animal-likeness and likability [6]. At the time of that study, only a limited number of drones were designed with animal-like features. Compared to their database, ours includes more animal-like drones, and through our likability-based clusters characterization, we find that **the type of animal representation influences likability**. Drones designed as bugs with negative connotations (e.g., spiders and insects) are clustered in the non-likable cluster, while drones designed as insects with positive connotations (e.g., butterflies, ladybugs, or bees) are clustered in the neutral and likable clusters.

Prior literature suggested that a social drone should have a face [34] and facial features [6,35], and that emotional expressions emotionally affect users [29]. Our results show that **the emotional expression type impacts likability**. Drones with facial features presenting emotional expressions of joy or sadness were clustered in the neutral and likable clusters, while drones with emotional expressions of anger were clustered as non-likable.

Our findings also corroborate prior research [6] and show that **likable drones are characterized by a visible camera without propeller guards**, compared to the other clusters of neutral and non-likable.

## 6. Limitations and Future Work

The main limitation of this work is the dataset size. While many drone images are available online via search engines, building a database for research purposes requires identifying images with specific characteristics. For instance, the database used in our work is composed of drone images with characteristics such as: propellers at rest, i.e., not flying; light unicolor background; no identifying logos; etc. Moreover, prior work suggests that “a few drone manufacturers sell the same model to companies who then perform light customization” so that many drones on the market present highly similar visual features [6]. Given this trend and the current global economic climate, we expect that much time needs to pass before this type of database can grow. As such, more future work is needed focusing on computational methods for small datasets. In addition, in this work, we chose to focus on likability. Future work could investigate other drone ratings, such as friendliness, trustworthiness, or even intelligence [6], which may lead to different clusters and new insights for future designs and research.

## 7. Conclusions

With recent advances in DL, and specifically GANs, for synthetic image generation, we proposed to apply such methods for application in HDI research. This work in particular aimed at generating novel synthetic images of likable drones for future social applications. This paper described the method for collecting drone images in a database applicable for drone design research. It then presents the results of a user study (N=379) identifying how people perceive drones in terms of likability. We further described two clustering methodologies: likability-based, employing the likability ratings, and feature-based, employing a pre-trained VGG16 network for image feature extraction and PCA for dimensionality reduction. Both methodologies operate data clustering using the K-means algorithm. Through a characterization process, we identified three drone features: colorfulness, animal-like representation, and emotional expressions through facial features, which affect the likability of drones, validating and going beyond prior findings in HDI. We focused on the cluster of likable drones for generating new images using StyleGAN2-ADA. We considered our data limitations (122 images) and experimented with several configurations of parameters and five pre-trained networks for transfer learning. We further reported results showing that the cluster of likable drones is linked to the color blue. We then illustrated the feasibility of our approach by generating new valid drone designs based on the original drone database (360 images). These findings can support drone designers, manufacturers, and researchers in a fully automated workflow for future drone designs without human intervention. This research contributes to: 1. understanding that computational methods are effective for categorizing drone images based on human perception; 2. identifying three visual features as markers of likability beyond prior work; and 3. new drone designs can be automatically generated using GANs. To the best of our knowledge, this work is the first to exploit DL-based methodologies and techniques for HDI research in the context of drone design.

## Figures and Tables

**Table 1:** KID value for each configuration of transfer learning variant and the corresponding time elapsed until an optimal value. A lower KID value is better for GANs. The CelebA-HQ-30k pre-trained network results best for our data compared to the other pre-trained configurations and the baseline configuration training from scratch.

Transfer Learning Variant	KID $\times \;10^{3}$	Time (h)
Baseline (from scratch)	51.46	60
**CelebA-HQ-30k**	**23.16**	68
LSUNCAT-1k	46.26	33
LSUNCAT-200k	39.74	51
FFHQ-10k	41.32	31
FFHQ-140k	29.87	47

## Data Availability

The data presented in this study will be made available upon request to the authors.

## Abbreviations

The following abbreviations are used in this manuscript:

ADA	Adaptive Discriminator Augmentation
CV	Computer Vision
DCGAN	Deep Convolutional GAN
DL	Deep Learning
FID	Fréchet Inception Distance
GAN	Generative Adversarial Network
HDI	Human-Drone Interaction
KID	Kernel Inception Distance
ML	Machine Learning
PCA	Principal Component Analysis
SSE	Sum Squared Error
VGG	Visual Geometry Group
